# True or false coral snake: is it worth the risk? A *Micrurus corallinus* case report

**DOI:** 10.1186/s40409-018-0148-9

**Published:** 2018-04-10

**Authors:** Marcelo Abrahão Strauch, Guilherme Jones Souza, Jordana Nahar Pereira, Tyelli dos Santos Ramos, Marcelo Oliveira Cesar, Marcelo Amorim Tomaz, Marcos Monteiro-Machado, Fernando Chagas Patrão-Neto, Paulo A. Melo

**Affiliations:** 1 Vital Brazil Institute, Niterói, RJ Brazil; 2 Laboratory of Pharmacology of Toxins, Institute of Biomedical Sciences, Center of Health Sciences, Federal University of Rio de Janeiro, Rio de Janeiro, RJ Brazil; 3 Hospital das Clínicas Constantino Ottaviano, Teresópolis, RJ Brazil; 4 Postgraduate Program in Teaching of Science, Environment and Society, Rio de Janeiro State University, Rio de Janeiro, RJ Brazil

**Keywords:** Coral snake, Envenoming, *Micrurus* spp*.*, Snakebites, Ophidism

## Abstract

**Background:**

Bites provoked by the genus *Micrurus* represent less than 1% of snakebite cases notified in Brazil, a tiny fraction compared with other genus such as *Bothrops* and *Crotalus*, which together represent almost 80% of accidents. In addition to their less aggressive behavior, habits and morphology of coral snakes are determinant factors for such low incidence of accidents. Although *Micrurus* bites are rare, victims must be rescued and hospitalized in a short period of time, because this type of envenoming may evolve to a progressive muscle weakness and acute respiratory failure.

**Case Presentation:**

We report an accident caused by *Micrurus corallinus* involving a 28-year-old Caucasian sailor man bitten on the hand. The accident occurred in a recreational camp because people believed the snake was not venomous. The victim presented neurological symptoms 2 h after the accident and was taken to the hospital, where he received antielapidic serum 10 h after the bite. After the antivenom treatment, the patient presented clinical evolution without complications and was discharged 4 days later.

**Conclusions:**

We reinforce that it is essential to have a health care structure suitable for the treatment of snakebite. Besides, the manipulation of these animals should only be carried out by a team of well-equipped and trained professionals, and even so with special attention.

## Background

Most tropical countries face morbidity and mortality induced by animal envenomation as a group of neglected diseases. Incidence of snakebites is over two million cases per year worldwide, with approximately 100,000 deaths, and non-estimated transient or permanent sequelae [1–4]. In Brazil, official data report approximately 27,000 accidents involving snakes per year, most of which caused by pit vipers (*Bothrops,* 71.41%), rattlesnakes (*Crotalus,* 7.03%), bushmasters (*Lachesis,* 3%) and true corals (*Micrurus,* 0.78%) [5, 6].

**Fig. 1:**
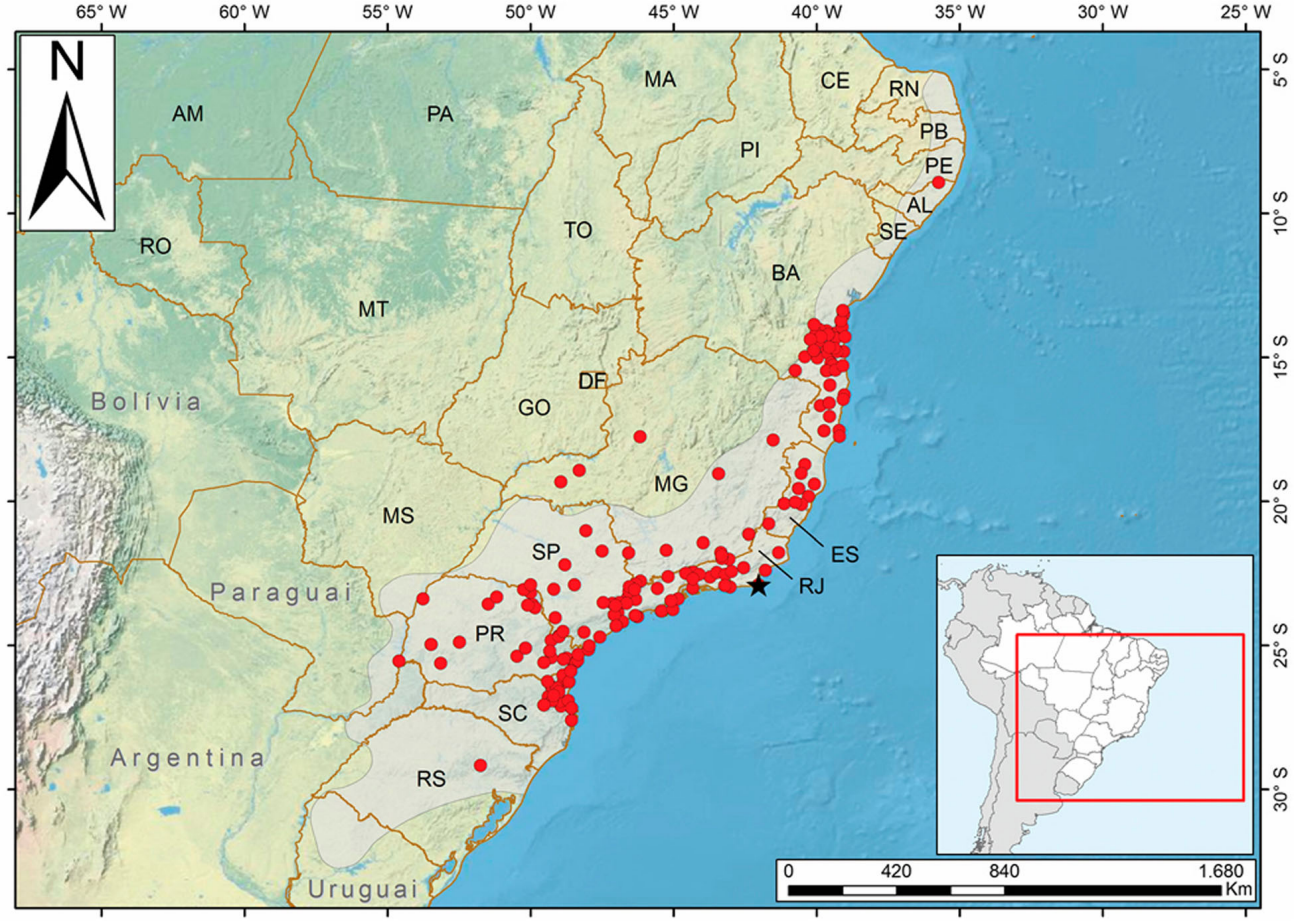
Map showing the Atlantic Forest area on the Brazilian coast where *Micrurus corallinus* is found [7]. Copyright by Prof. Nelson Jorge da Silva Jr. Reprinted with permission

The genus *Micrurus* (the so-called true coral snakes) has a wide geographic distribution in Brazil. *Micrurus corallinus* and *Micrurus frontalis* are the two most frequent species, and Fig. 1 shows the distribution of the former in Brazilian coast [7]. *Micrurus* spp. have small to medium size, with short maxillary fangs (proteroglyph dentition) and are characterized by marked and attractive reddish coloration, presenting complete black, yellow or white rings around the body [8, 9]. The venom of these snakes generally present neurotoxic, myotoxic, nephrotoxic, hemorrhagic and edematogenic activities. Moreover, neuromuscular blockade comprises the systemic hallmark of envenoming by *Micrurus* spp. [10]. Due to resembling colors and rings, they are commonly mistaken for non-venomous snakes of families Dipsadidae, Colubridae and Aniliidae, altogether referred to as false coral snakes [10].

Coral snakes are generally not aggressive or prone to biting, and when confronted by humans they will most likely attempt to flee, so they bite only as a last resort [11]. Although uncommon, envenomation by *Micrurus* spp. in humans should always be considered serious. Therefore, public health systems ought to regard the antielapidic serum (AES) availability and administration, and its efficacy in neutralizing the venom toxins. Many people may have already heard: when facing a snake that resembles a coral snake, do not hesitate in considering it a true coral. Inadvertent manipulation of coral snakes when considering the animal to be a non-venomous specimen accounts for many cases of envenomation, which poses the question: is it worth to handle coral snakes unnecessarily? In this case report, we aim to describe such a case of envenoming by *Micrurus corallinus* that occurred in the city of Cachoeiras de Macacu, RJ, Brazil.

## Case presentation

**Fig. 2:**
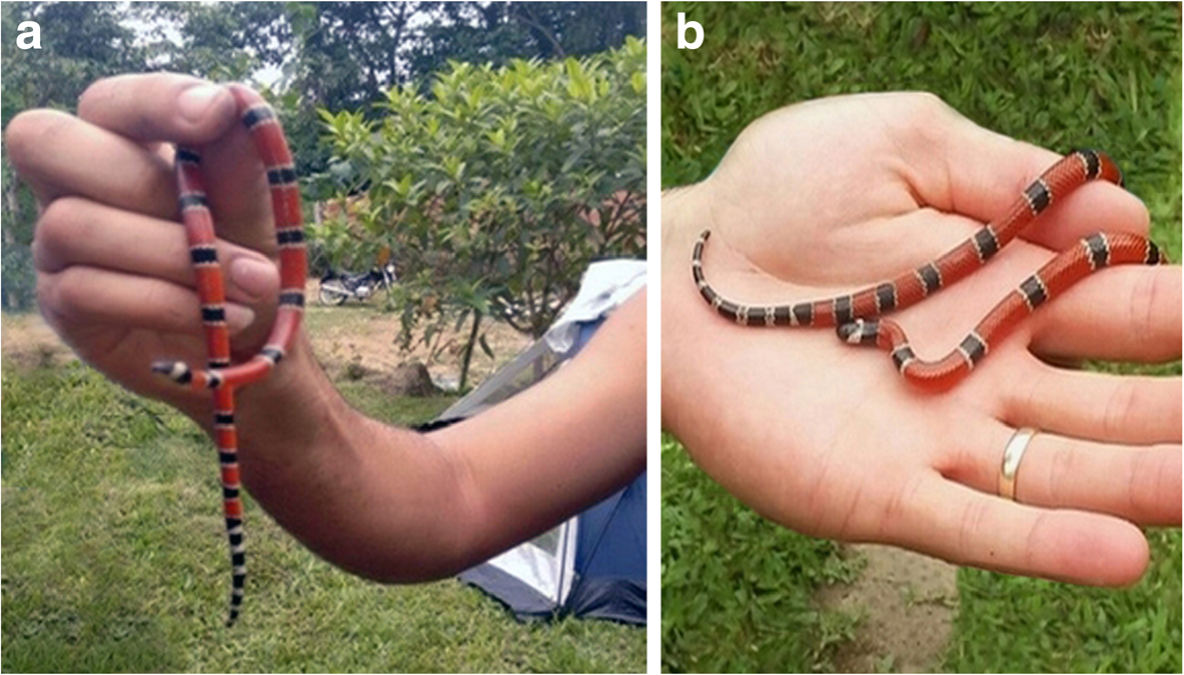
Handling of a venomous coral snake, without use of suitable equipment

A 28-year-old Caucasian sailor man, born and living in Cachoeiras de Macacu, RJ, was in a recreational camp with a group of friends when he spotted a snake on the ground. A biologist present on the camp captured the animal with his bare hands believing it was a false coral snake (Fig. 2). The snake was photographed and imprudently manipulated by many people in the camp, including the patient, who was bitten on the back of the right hand, between the thumb and index finger around 1:00 p.m.

A few minutes later, the patient complained of mild local pain. During the afternoon, due to the beginning of visual disturbances, the friends of the victim took him to the city hospital, 20 km away from the camp. On admission, 6 h after the accident, he received 1000 mL of 0.9% NaCl saline solution and 500 mg hydrocortisone intravenously. Since the health unit did not have antielapidic serum (AES), the patient was transferred to the Hospital das Clínicas Constantino Ottaviano, in the city of Teresópolis, RJ, where he arrived around 8 h following the accident.

**Fig. 3:**
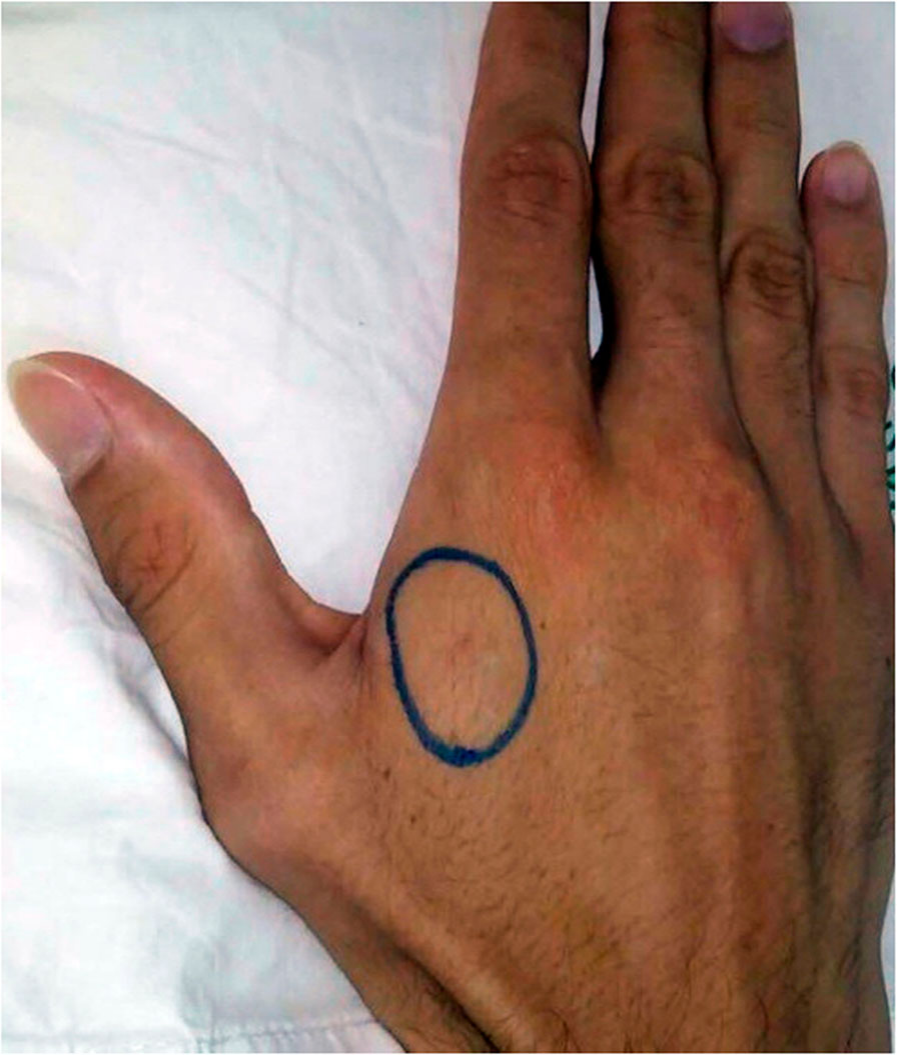
Snakebite site on the right hand

At that moment, the patient was presenting neurotoxic manifestations such as bilateral palpebral ptosis, diplopia, dysphagia and tongue paresthesia, in addition to low back pain. Vital signs were all within normal range (body temperature 36.7 °C, blood pressure 110/70 mmHg, pulse 78 bpm, and breathing rate 16 bpm). On the bite site (Fig. 3), there were no signs of local damage, such as edema or hyperemia. The patient received 100 mL of AES, divided into two doses of 50 mL each. Five vials (10 mL each) were sent from the Sul Fluminense University Hospital, Vassouras, RJ (140 km away) and the other five vials were from the Oswaldo Cruz Foundation, FioCruz, Rio de Janeiro, RJ (95 km away).

The two steps of serum therapy were diluted in 0.9% NaCl saline solution and infused intravenously in 40 min without any adverse reactions. The patient was then admitted at the intensive care unit for 1 day for close cardiovascular and respiratory monitoring, and two more days in in-patient care unit, where he evolved with clinical improvement. Laboratory tests were performed during 3 days of hospitalization (Table 1), and showed no major or specific alterations, except for a small unspecific rise in plasma creatine kinase (CK) activity. The patient remained asymptomatic and was discharged on the fourth day of hospitalization.

**Table 1:** Results of laboratory tests carried out during hospitalization

Laboratory tests	Day 1	Day 2	Day 3	References
Red blood cells	5.45	5.89	4.73	4.5–5.5 10^6^/mm^3^
Hematocrit	44.9	43.2	38.7	40–55%
Hemoglobin	14.9	15.7	13.0	12–17
Leucocytes	8.9	9.9	9.9	5–10 × 10^3^/mm^3^
Basophils	0	0	0	0–1%
Eosinophils	1	1	0	2–5%
Myelocytes	0	0	0	0%
Metamyelocytes	0	0	0	0%
Band neutrophils	5	5	2	3–5%
Segmented neutrophils	80	80	78	55–66%
Lymphocytes	13	13	14	20–35%
Monocytes	1	1	6	4–8%
Platelets	212	227	–	150–450 × 10^3^/ mm^3^
Clotting time	9.0	5.30	–	8–15 min
Bleeding time	1.30	3.20	–	2–7 min
Blood urea nitrogen	28	24	33	10–50 mg/dL
Creatinine	0.7	0.7	0.9	0.7–1.5 mg/dL
Sodium	138	139	141	135–145 mEq/L
Potassium	4.0	4.0	3.4	3.5–4.5 mEq/L
CK	–	374	489	26–189 IU/L

## Discussion

The report of this case aims to raise some questions, the most important of which is: is it worth to classify a coral snake as non-venomous, even if you are a specialist? The Brazilian literature [6, 10, 12, 13] describes accidents with coral snakes as rare, even when cases with non-venomous species are considered. Due to their non-aggressive behavior, most accidents involving coral snakes are the result of incorrect or reckless handling of these snakes, so that hands and fingers are more frequently affected [14–16]. Elapid envenoming is a public health problem in several regions of the world. The high diversity of *Micrurus* species in Brazil should raise concern for human accidents, because genomic variations in individuals of different species within the same genus are significant and relevant, especially when we analyze the differences in the proteins that compose the venoms [17, 18]. Such variations directly affect the immunogenicity and the different physiological changes caused in cases of envenoming.

*Micrurus* venoms have several pharmacological actions, the most common being neurotoxic, myotoxic, edematogenic and hemorrhagic [10]. Remarkable presynaptic actions of phospholipases A_2_ or postsynaptic effects of three-finger toxins (3FTx) provoke neuromuscular blockage, with variable effects in contraction capacity and muscle strength. Intense muscular paralysis reaching diaphragm is the cause of death in cases of expressive *Micrurus* envenomation [19, 20]. Some previous observations described that some coral snake venoms are able to damage skeletal muscle fibers and induce myonecrosis [21]. In the present case, plasma CK activity did not change significantly to suggest relevant myotoxic activity, which, besides the limited neuromuscular effect observed, indicates that the amount of venom (e.g., snake age) or the size of the victim are important in the observed outcomes.

Officially, AES in Brazil is manufactured by the Butantan Institute in São Paulo, and by the Ezequiel Dias Foundation in Minas Gerais. It is produced based on the venoms of *M. corallinus* and *M. frontalis*. These species are relatively common in populous regions in Brazil, such as South and Southeast, which facilitates the collection of specimens to obtain the venom (Fig. 1). However, studies with venoms from different *Micrurus* species have shown that they present a variety of toxins in their composition. For example, *M. corallinus* venom presents higher amounts of PLA_2_ than 3FTx [22], which could diminish or even significantly limit the protective capacity of the standard AES when used in accidents caused by different species of snakes of this genus [19, 21, 23, 24].

Another relevant issue is the distribution of specific antivenom. Snakebites are on the World Health Organization list of neglected tropical diseases. Due to the lack of knowledge on the biological, clinical and epidemiological aspects related to the problem, accidents with venomous snakes do not generally count with a structured and diffuse plan of assistance [25]. According to Brazilian Ministry of Health, elapid accidents that present neurological symptoms are characterized as potentially serious, so that serum therapy is strongly recommended, using 10 vials (100 mL) of AES administered intravenously in a single dose [26]. The treatment described in this reported case was inappropriate since the serum therapy was divided into two doses of 50 mL due to the lack of the whole dose in the hospital. Moreover, administration of corticosteroids, although not strictly contraindicated, is not part of the primary approach to elapid envenomation. The patient stayed in the hospital long enough to avoid venom redistribution from the snakebite site [27].

Finally, we would like to emphasize this issue: why expose yourself to the risk of touching or handling a coral snake by judging it as non-venomous? In North America, there is a popular rhyme that many people know that has for decades been a popular way of telling them apart: “red-on-yellow, kill a fellow” and “red-on-black, venom lack.” The idea is that “true” coral snakes can be identified by red bands touching the yellow ones. It may be helpful in telling coral snakes apart from non-venomous species in the USA, for example, but the color pattern is not the same for all *Micrurus* species. Therefore, the rhyme rule cannot be considered reliable in all South America, for instance, nor for all species, even though the rule could be correctly applied to the case herein described. Therefore, unless this is part of your work (e.g. as a biologist or herpetologist), and especially if you are in a recreational camp with a group of friends, it is safer to consider the Brazilian rule of thumb: “when you spot a coral snake, always consider it to be a true one”.

## Conclusions

The care and treatment provided by the medical team in Hospital das Clínicas Constantino Ottaviano was satisfactory to revert the clinical picture of the envenoming, even with serum therapy performed in two steps, and following corticosteroid injection. In face of the risk to human health, due to potentially serious neurotoxicity, it is essential to guide professionals so that occupational accidents could be minimized with the use of proper equipment, and, especially, to educate the general population on the subject.

## Abbreviations

3FTx: three-finger toxins
AES: antielapidic serum
CK: creatine kinase

## References

[1] Chippaux JP (1998). Snake-bites: appraisal of the global situation. Bull World Health Organ.

[2] Williams D, Gutiérrez JM, Harrison R, Warrell DA, White J, Winkel KD (2010). The global snake bite initiative: an antidote for snake bite. Lancet.

[3] Gutiérrez JM, Calvete JJ, Habib AG, Harrison RA, Williams DJ, Warrell DA (2017). Snakebite envenoming. Nature.

[4] Chippaux JP (2017). Snakebite envenomation turns again into a neglected tropical disease!. J Venom Anim Toxins incl Trop Dis.

[5] Brasil. Secretaria de Vigilância em Saúde. Sistema de Informação de Agravos de notificação (SINAN). Brasília. Ministério da Saúde do Brasil. 2017. http://tabnet.datasus.gov.br/cgi/tabcgi.exe?sinannet/cnv/animaisbr.def. Accessed 14 Aug 2017.

[6] Chippaux JP (2015). Epidemiology of envenomations by terrestrial venomous animals in Brazil based on case reporting: from obvious facts to contingencies. J Venom Anim Toxins incl Trop Dis..

[7] Silva Junior NJ, Pires M, Feitosa D. Diversidade das Cobras Corais do Brasil. In: Silva Junior NJ editor. As Cobras Corais do Brasil. 1ª ed. Goiás: PUC; 2016. p. 96.

[8] Di-Bernardo M, Borges-Martins M, Silva NJ (2007). A new species of coralsnake (*Micrurus*: Elapidae) from southern Brazil. Zootaxa.

[9] Ernst CH, Ernst EM (2011). Venomous reptiles of the United States, Canada and Northen Mexico: *Heloderma*, *Micruroides*, *Micrurus*, *Pelamis*, *Agkistrodon*, *Sistrurus*. Johns Hopkins University Press.

[10] Bucaretchi F, Capitani EM, Vieira RJ, Rodrigues CK, Zannin M, Da Silva NJ (2016). Coral snake bites (*Micrurus* spp.) in Brazil: a review of literature reports. Clin Toxicol (Phila).

[11] Sanz L, Pla D, Pérez A, Rodríguez Y, Zavaleta A, Salas M (2016). Venomic analysis of the poorly studied desert coral snake, *Micrurus tschudii tschudii*, supports the 3FTx/PLA2 dichotomy across *Micrurus* venoms. Toxins (Basel).

[12] Coelho LK, Silva E, Espositto C, Zanin M (1992). Clinical features and treatment of Elapidae bites: report of three cases. Hum Exp Toxicol.

[13] Cañas CA, Castro-Herrera F, Castaño-Valencia S (2017). Envenomation by the red-tailed coral snake (*Micrurus mipartitus*) in Colombia. J Venom Anim Toxins incl Trop Dis..

[14] McCollough NC, Gennaro JP (1963). Coral snake bites in the United States. J Fla Med Assoc.

[15] Parrish HM, Khan MS (1967). Bites by coral snakes: report of 11 representative cases. Am J Med Sci.

[16] Kitchens CS, Van Mierop LHS (1987). Envenomation by the eastern coral snake (*Micrurus fulvius fulvius*). A study of 39 victims. JAMA.

[17] Tan NH, Ponnudurai G (1992). The biological properties of venoms of some American coral snakes (genus *Micrurus*). Comp Biochem Physiol B.

[18] Jorge da Silva N, Aird SD (2001). Prey specificity, comparative lethality and compositional differences of coral snake venoms. Comp Biochem Physiol C Toxicol Pharmacol.

[19] Moreira KG, Prates MV, Andrade FAC, Silva LP, Beirão PSL, Kushmerick C (2010). Frontoxins, three-finger toxins from *Micrurus frontalis* venom, decrease miniature endplate potential amplitude at frog neuromuscular junction. Toxicon.

[20] Corrêa-Netto C, Junqueira de Azevedo ILM, Silva DA, Ho PL, Leitão-de-Araújo M, Alves MLM (2011). Snake venomics and venom gland transcriptomic analysis of Brazilian coral snakes, *Micrurus altirostris* and *M. corallinus*. J Proteome.

[21] Gutiérrez JM, Rojas G, da Silva NJ, Núñez J (1992). Experimental myonecrosis induced by the venoms of south American *Micrurus* (coral snakes). Toxicon.

[22] Aird SD, da Silva NJ, Qiu L, Villar-Briones A, Saddi VA, Pires de Campos Telles M, et al. Coralsnake venomics: analyses of venom gland transcriptomes and proteomes of six brazilian taxa. Toxins (Basel). 2017;9(6):187. 10.3390/toxins9060187 PMC5488037 28594382

[23] Tanaka GD, Furtado Mde FD, Portaro FCV, Sant’Anna OA, Tambourgi DV (2010). Diversity of *Micrurus* snake species related to their venom toxic effects and the prospective of antivenom neutralization. PLoS Negl Trop Dis.

[24] Ciscotto PH, Rates B, Silva DA, Richardson M, Silva LP, Andrade H (2011). Venomic analysis and evaluation of antivenom cross-reactivity of south American *Micrurus* species. J Proteome.

[25] Gutiérrez JM, Theakston RDG, Warrel DA (2006). Confronting the neglected problem of snake bite envenoming: the need for a global partnership. PLoS Med.

[26] Brasil. Ministério da Saúde. Fundação Nacional de Saúde. Manual de Diagnóstico e Tratamento de Acidentes por Animais Peçonhentos. 2ª ed. 2001. 120 p. http://bvsms.saude.gov.br/bvs/publicacoes/funasa/manu_peconhentos.pdf. Accessed 14 Aug 2017.

[27] Vergara I, Castillo EY, Romero-Piña ME, Torres-Viquez I, Paniagua D, Boyer LV (2016). Biodistribution and lymphatic tracking of the main neurotoxin of *Micrurus fulvius* venom by molecular imaging. Toxins (Basel)..

